# Intrauterine fetal death in triplet gestation caused by feto-fetal transfusion syndrome – a case report

**DOI:** 10.1080/20961790.2016.1264915

**Published:** 2016-12-16

**Authors:** Lingling Long, Jie Yan, Qiyan Li, Ziqi Zhou, Haixiao Deng, Chudong Wang, Ying Zou, Jifeng Cai

**Affiliations:** a Department of Forensic Science, School of Basic Medical Science, Central South University, Changsha, China; b Department of Forensic Pathology, Hunan Xiangya Judicial Identification Center, Changsha, China

**Keywords:** Feto-fetal transfusion syndrome, monochorionic triplet pregnancy, vascular anastomoses, intrauterine fetal death, obstetrics

## Abstract

Feto-fetal transfusion syndrome (FFTS) severely affects monochorionic (MC) multiple pregnancies and affects 1 in 1600 pregnancies overall. The number of increasing disputed obstetrics cases in China is related to unavailability of prompt diagnosis of FFTS. We present here a woman with a MC triplet pregnancy with intrauterine fetal death at 33 weeks of gestation due to FFTS. Subsequent pathological anatomy showed that the MC placenta contained vascular anastomoses, including arterio-arterial anastomosis and arterio-venous anastomosis. These anastomoses led to unidirectional blood flow with the absence of adequate compensatory counter-transfusion and bi-directional flow. When encountering such challenging conditions, medical practitioners should discreetly compare the fetuses’ characteristics with features of placental blood vessels and consult morphological and pathological findings. Furthermore, they should perform ultrasound examinations, particularly focussing on fetal size differences and the maximum vertical pocket in the diagnosis of FFTS, especially in MC multiple pregnancies with abdominal symptoms.

## Introduction

Feto-fetal transfusion syndrome (FFTS) occurs in approximately 15% of monochorionic (MC) multiple gestations with MC placentation [1]. FFTS leads to adverse outcomes, such as twin-to-twin transfusion syndrome (TTTS), a complex and serious cardiovascular clinical disease affecting MC twin pregnancies. TTTS triggers development of severe oligohydramnios and hypoxia in the donor fetus and polyhydramnios and cardiac failure in the recipient fetus [2]. TTTS accounts for approximately half of all perinatal death associated with MC twin pregnancy [3]. Additionally, the absence of medical treatment contributes to extremely high perinatal mortality and morbidity of greater than 80% [4].

Cases of TTTS are more common than triplet-to-triplet transfusion syndrome [5]. We present a case of a combination of MC triplet pregnancy with FFTS resulting in co-triplet fetal demise. This seldom occurring case will be of great value in the settlement of difficult medical disputes involving fatal death in China.

## Case report

A 29-year-old woman, gravida 2, para 0, was hospitalized for abdominal pain in the lower part of the abdomen with no apparent cause at 32^+2^ weeks of gestation. Ultrasound showed MC triplet gestation with a difference in fetal weight estimates (two fetuses at 32 weeks and the other at 31 weeks). Placental function ranged between grades I and II. The umbilical cord of one fetus was once around the neck. Umbilical artery Doppler and a non-stress test showed no unusual symptoms. No distinct evidence of FFTS was observed during the entire pregnancy. After the woman was diagnosed with threatened premature delivery, she was treated with phloroglucinol for tocolysis and dexamethasone for promoting lung maturation of the three fetuses. The next day, the woman complained of continuous abdominal distension, while her previous abdominal pain was relieved. An ultrasound examination on the fourth day showed two fetuses with a weight estimated for 33 weeks and the other for 32 weeks of gestation. The non-stress test indicated slight abnormality. Therefore, conservative medical care was continuously implemented. At 4:30 am on the fifth day, the three fetal heartbeats stopped and fetal movement ceased. Thereafter, the three deceased fetuses weighted 1700, 2100 and 1800 g when they were delivered by caesarean section.

**Figure 1.**
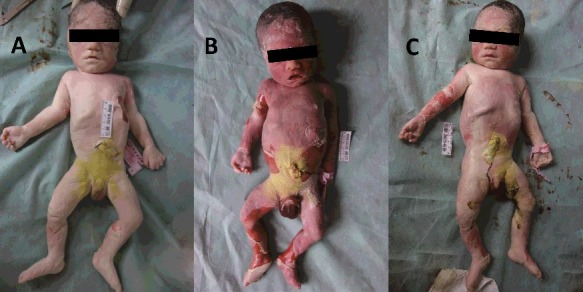
Both the fetuses A and C were thin and pale while the fetus B was heavy and red.

**Figure 2.**
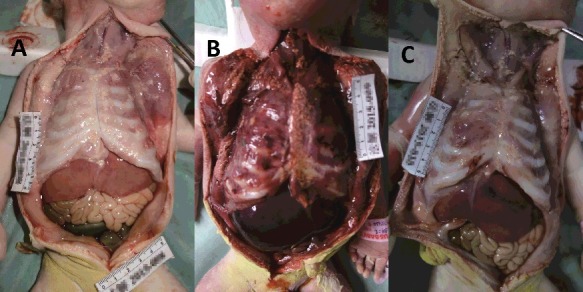
Internal organs showed that fetuses A and C presented anaemia changes (A and C). The fetus B performed congestion and haemorrhage of organs (B).

An external examination showed that fetuses A and C were thin and pale, while fetus B was heavy and red (Figure 1). Observation of the internal organs showed that fetuses A and C suffered anaemic changes (Figure 2(A,C)). However, fetus B presented congestion and haemorrhage of the organs (Figure 2(B)).

**Figure 3.**
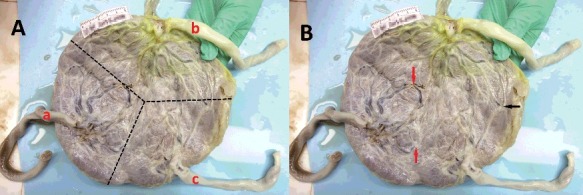
(A) The umbilical cord of recipient's (a) was dark while that of donor's (b and c) were pale. The hatched lines indicated relatively symmetrical triplet placenta portions of the single placental disc. (B) There were two types of anastomosis in MC placenta: the superficial AAA (red arrow) and deep AVA (black arrow).

Examination of the MC placenta indicated that the umbilical cord of the recipient performed differently with the donor (Figure 3(A)). All three fetuses shared relatively symmetrical triple placental portions of the single placental disc. Two types of vascular anastomoses, including arterio-arterial anastomosis (AAA) and arterio-venous anastomosis (AVA), were observed in the placenta (Figure 3(B)).

**Figure 4.**
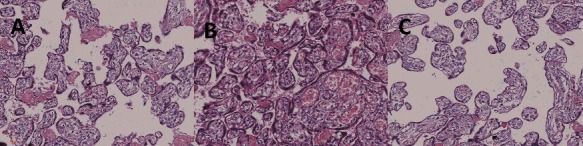
(A and C) Anaemia area of the placenta: relatively large immature villi and interstitial edema. (B) Hyperaemia area of the placenta: villi were mature with congestive interstitial telangiectasia. Syncytiotrophoblast nucleus degenerated, concentrated and gathered into multi-core nodules.

Microscopic examination of placental villi showed differences in anaemia and hyperaemia. We observed relatively large and immature villi with interstitial oedema in the anaemic area of the placenta (Figure 4(A,C)). In the hyperaemic area of the placenta, villi were mature with congestive interstitial telangiectasia. Furthermore, syncytiotrophoblast nuclei were degenerated, concentrated and gathered into multi-core nodules (Figure 4(B)).

Laboratory studies showed that haemoglobin values of the fetuses were 58, 135 and 65 g/L for fetuses A, B and C, respectively.

## Discussion

Triplet pregnancies with FFTS are a rare syndrome classified as MC placentation. There are inadequate related reports in the literature of this condition. Therefore, we are limited in comparative analysis. FFTS is a potential complication of multiple gestations, and is a major determinant of morbidity and mortality [6]. FFTS occurs and evolves at different gestational ages. Therefore, some affected gravidas display a quick course, while others demonstrate a more stable process. So it is relatively difficult for the accurate evaluation of the degree of clinical variation and restricted in precisely predicting the procession of FFTS.

Intertwin transfusion likely leads to FFTS, and the conclusion that most MC placentas contain vascular anastomoses has been confirmed by *ex vivo* dye injection [7]. There are three types of interplacental anastomoses, including arterio-arterial, veno-venous and arterio-venous. Further, all of the anastomoses can be divided into two groups of superficial and deep anastomoses [8]. Superficial anastomoses refer to true linking between the same type of chorionic vessels as AAA and venous--venous anastomosis (VVA), along with mediating blood flow bi-directionally. A deep anastomosis, known as an AVA, mediates transfusion beneath the chorionic plate within a cotyledon, coupled with unidirectional flow between the two circulations as artery to vein. A deep anastomosis is not an exact or typical one, despite an AVA connecting an artery with a vein, because an AVA does not bypass the capillary circulation [5]. A higher risk for development of TTTS is associated with the presence of AVAs without compensating AAAs [9]. AAAs are present in 90%–95% of MC placentas, AVAs in 85%–90% and VVAs in only 15%–22% [9–11].

After primary hemodynamic discordance created by placental anastomotic transfusion, the donor becomes hypovolemic and oliguric. However, the recipient becomes hypervolaemic and polyuric if the shift in blood flow becomes large. The renal system of the donor activates the renin–angiotensin system, resulting in hypertension [12]. Hypervolaemia syndrome of the recipient largely relies on phenotypic features. High atrial natriuretic peptide levels, secreted in response to fluid overload, along with concomitant suppression of antidiuretic hormone, mediate the associated polyuria and polyhydramnios [13]. Volume overload in the recipient causes cardiovascular findings, such as atrioventricular valve regurgitation, ventricular hypertrophy and increased aortic outflow and pulmonary outflow velocity [14]. As a result, the imbalance of blood transfusion initiates a cascade of events in the fetuses, leading to significant morbidity and mortality associated with untreated TTTS.

FFTS is diagnosed prenatally by ultrasound when there is at least one fetus with oligohydramnios or one fetus with polyhydramnios. The diagnostic criteria of FFTS in MC triamniotic triple gestations are similar to those of twin gestations. By ultrasound, TTTS is defined as follows: (1) MC multiple gestations; (2) the presence of polyhydramnios (maximum vertical pocket of ≥8 cm) and oligohydramnios (maximum vertical pocket of ≤2 cm) [15]. In the absence of ultrasound, FFTS in MC pregnancies is diagnosed based on growth discordance of 20% associated with a discordant fetal haemoglobin level of 50 g/L [4]. Moreover, the placenta is extraordinarily valuable for diagnosing FFTS. The sensitive technique of vascular casting and *ex vivo* injection can identify and confirm atypical AVA hidden beneath the chorionic plate, which would remain unnoticed via conventional inspection. Slaghekke et al. [16] provided a simple protocol to accurately evaluate the presence of (residual) vascular anastomoses using coloured dye injection.

Precise prenatal diagnosis and intervention treatment play vital roles because of the high risk of morbidity and mortality of FFTS. The current treatments of FFTS include amnioreduction, septostomy and fetoscopic laser photocoagulation. Fetoscopic laser photocoagulation is considered the optimal therapy for FFTS that presents before 26 weeks of gestation [17].

Compared with TTTS, only limited cases of FFTS with MC triplet pregnancies have been reported [2]. Findings in our case suggested that the three fetuses died of FFTS as indicated by the following pathological characteristics: dramatically distinct presentation of the skin, organs and villi; growth discordance; a difference in haemoglobin values among the fetuses. Triplet-to-triplet transfusion syndrome may progress according to two models determined by the type of placental vascular anastomoses: one donor donates blood to two recipients or one recipient receives blood from two donors. The latter situation is presented in this case. In forensic practice, ultrasound evidence of MC triplet pregnancies and abnormal blood transfusion are important. Additionally, a general post-mortem examination containing differences in skin colour and discordance of weight, as well as laboratory studies of haemoglobin, are important in forensic practice. Furthermore, pathological examinations show ischaemia and hypoxia of the donor's organs compared with congestion and oedema of the recipient. Specific attention to the placental vessels should also be given. If vascular anastomoses are present, especially AVAs, in the placenta, diagnosis of FFTS can be determined.

Obstetrics disputes are becoming one of the most disconcerting concerns of medical lawsuits because of the high morbidity and mortality of two vulnerable generations: mothers and neonates. This is especially the case in China, with decades of a one-child national policy [18]. Medical negligence in obstetrics can occur during any of the following three processes of medicare: diagnosis, advice and treatment. In our case, the clinical diagnosis of FFTS was neglected during hospitalization, despite signs of a MC triplet pregnancy, abdominal pain, distension and difference in weight estimates. Even an important diagnostic factor of FFTS, such as the maximum vertical pocket, was not measured. Therefore, no intervention targeting FFTS was performed in time. Additionally, the high cost of treatment of a possibly poorly developed neonate by premature delivery may be another crucial consideration for doctors and patients, resulting in deliberation and delay of a caesarean operation. Therefore, to make a prompt and accurate diagnosis and reduce medical disputes, there are several aspects that could be improved in modern obstetrics. These aspects include continuing medical education, training and re-training of obstetricians and emphasizing obstetrics protocol, especially medical insurance coverage for obstetricians and patients.

## Conclusion

We report a rare case of MC triplet pregnancy with FFTS. Detailed pathological studies on MC triplet pregnancy with placental vascular anastomoses are essential for the diagnosis of FFTS. Furthermore, an ultrasound examination, including fetal size differences and the maximum vertical pocket, should be used for the diagnosis of FFTS, especially in the case of MC multiple pregnancies with abdominal symptoms.
